# Burden of migraine in Finland: health care resource use, sick-leaves and comorbidities in occupational health care

**DOI:** 10.1186/s10194-019-0964-5

**Published:** 2019-02-12

**Authors:** Minna A. Korolainen, Samu Kurki, Mariann I. Lassenius, Iiro Toppila, Madlaina Costa-Scharplatz, Timo Purmonen, Markku Nissilä

**Affiliations:** 1 Novartis Finland Oy, Espoo, Finland; 2 Terveystalo Biobank Finland, Humalistonkatu 7B, 20100 Turku, Finland; 3 Medaffcon Oy, Espoo, Finland; 4 Novartis AB, Kista, Sweden

**Keywords:** Migraine, Comorbidities, Prophylactic treatment, Treatment lines, Absenteeism, Disease burden

## Abstract

**Background:**

The highest prevalence of migraine is detected among people who are of working age. The aim of this study was to assess the burden of migraine in an occupational health care setting using real world data collected as a part of routine clinical practice.

**Methods:**

This retrospective register study included migraineurs using occupational health care at the private health care provider Terveystalo. An age and gender matched control population was established for comparison. Electronic medical records were assessed for overall and migraine related health care visits, sick-leaves and comorbidities. Stratification to acute and prophylactic treatment groups along with prophylactic treatment lines was based on prescriptions.

**Results:**

Among the 369,383 individuals in the study cohort, 7.4% women and 2.1% men were identified having a diagnosis of migraine. Prophylactic medication was prescribed to 13% of migraine patients and exclusively acute medication to 37%. Although migraine related visits and sick-leave days were significantly lower than overall visits or sick-leave days, both increased by prophylactic treatment line. The number of visits rose from 13.8 to 26.2 and sick-leave days from 16.8 to 30.4 per patient-year, in those without prophylaxis vs. ≥3 prophylactic treatments. Moreover, migraine patients had 1.7-fold increase in visits and 1.8-fold increase in sick leave days on average per patient-year, when compared to the control population. Depression and anxiety were 1.8-fold more common among patients with migraine, and the frequency also increase by treatment line.

**Conclusions:**

Migraine burden increased by each failed treatment line and was associated with increased comorbidity. In addition, migraine patients had significantly higher extent of visits and sick-leave days as well as extent of comorbidities when compared to their age- and gender-matched counterparts.

## Introduction

Migraine has a significant and debilitating impact on physical, social, and occupational functioning. In 2016, it was ranked the first cause of disability worldwide in both men and women under the age of 50 years highlighting the lack of effective disease management [1, 2].

Migraine is classified into episodic (EM) or chronic (CM) according to monthly migraine days. However, this classification is not unambiguous, and fluctuations between the two categories are evident from longitudinal studies [3]. Regardless of monthly migraine days, chronification of migraine is associated with substantially increasing individual and social burden. The burden of migraine has been elucidated in multiple surveys and studies [4–12] and recently Silberstein et al. showed that disease disability, health care resource use (HCRU), and direct costs increase concomitantly with increasing number of headache days [13]. Moreover, it has been established that the migraine burden builds up by increasing number of additional comorbidities such as psychiatric disorders, medication overuse, obesity, and respiratory and cardiovascular diseases [14–17]. It is known that e.g. depression and anxiety, as comorbidities among migraine patients, increase both direct and indirect costs [16, 18, 19].

Migraine is variably managed with acute (anti-inflammatory analgesics and triptans) and if needed with prophylactic (beta-blockers, tricyclics, candesartan and antiepileptics) treatment options [16, 20]. None of the current prophylactic treatments have originally been developed for migraine, and their specific mechanisms of action in migraine are not known. In a systematic review, adverse events were identified as the most common reason for treatment discontinuation [21]. In another study, up to 88% on prophylactic treatment reported lack of efficacy as the reason for treatment switch [22]. It is evident that adherence and persistence to current migraine prophylaxis is not optimal. The attention has recently thus turned into revealing the true impact of migraine disease burden also for patients in the need or failing prophylactic treatments [5, 23].

Several surveys and studies have been conducted on the burden of migraine, and some of them also using data from Finland [4, 24, 25]. However, studies on large register based data collected as part of clinical routine care are lacking. The main aim of the present study was to examine the burden of migraine and evaluate HCRU, sick-leaves and comorbidities among migraine patients compared to a control population in occupational health care setting.

## Methods

**Fig. 1:**
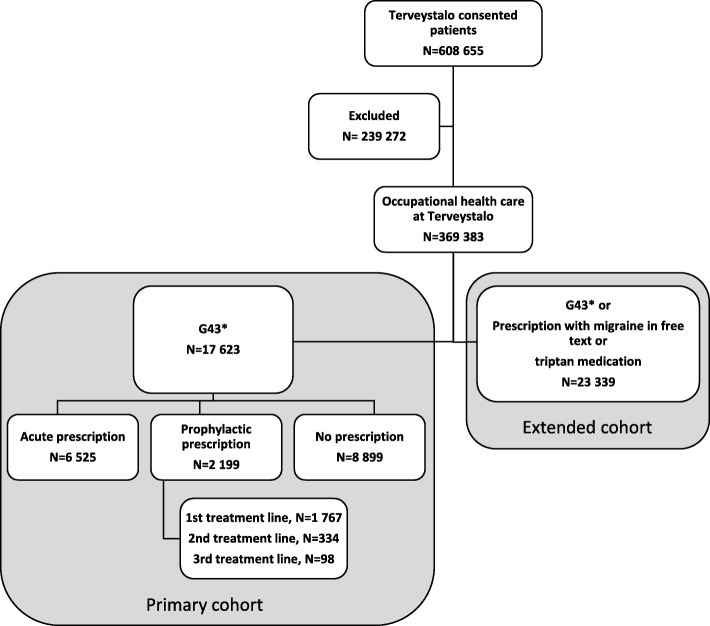
Study cohort formation

Electronic medical records (EMR) of a private health care provider Terveystalo were utilized in this retrospective register-based study. Patients who had given a written informed consent using occupational health care at Terveystalo (*N* = 369,383) were included (Fig. 1). The EMRs included diagnoses, visits, procedures, prescriptions, laboratory measures, and demographical characteristics. The data available covers outpatient care. Altogether, 17,623 of the patients had migraine according to ICD-10 code (G43*, on a three-character level) and were included in the primary cohort. All more detailed ICD-10 codes e.g. G43.5 were included to the three-character G43* level. To assess plausibly misdiagnosed migraine, patients were further included to an extended cohort based one of the following criteria: inclusion to the primary cohort (G43*) or a prescription with the word migraine in the drug prescription text, or prescribed triptan medication (*N* = 23,339, Fig. 1).

The inclusion period to the study and for the follow-up of patients started 1st Jan 2012 and ended 31st December 2017. Each patient was followed from the first G43* diagnosis in the EMR, or from the first fulfilment for any of the criteria for the extended cohort, until 31st December 2017.

Patients in the primary cohort were divided into groups based on medication prescribed at the health care provider. The following ATC classes were considered acute: M01-N02BE, N02CA, N02CC, A03FA, A04AA01, H02AB; and prophylactic: C07–09, N03, N06, G02–03, M03AX01 treatments. For both acute and prophylactic prescriptions, the migraine word in the free text of the prescription was required, since the same drugs can be prescribed for other illnesses as well. The division of patients to acute and prophylactic groups was validated by a neurologist. Botulinum toxin A is only indicated for CM in Finland, and these patients represent a confirmed CM population.

### Controls

A one-to-one, age and gender matched control population without migraine was created. For each migraine patient a control patient was randomly chosen based on gender and birth date from the database. No subjects were chosen twice for the control group (controls: *N* = 17,623, 76,804 patient-years, average age 38.9 years, 78.9% female; Primary cohort: N = 17,623, 51,396 patient-years, average age 38.9 years, 78.9% female). Controls were used as reference for comorbidity estimations, overall sick leaves and visits.

### Prevalence

In the health care provider’s register on 31st December 2017, the point prevalence for migraine was calculated by gender in 5-year age groups in the primary and extended cohorts. The consented occupational health care population was used as denominator for the calculation of prevalence.

### Treatment lines

A new treatment line was defined as a new unique prophylactic prescription in the health care provider’s register. Treatment lines were assessed for those with prophylactic prescriptions (later referred to as treatment lines). In addition to the word migraine in the free text of the prescription, a G43* diagnosis was required for the analyses. For a full list of prophylactic treatment lines see Appendix 1. For a list of acute migraine medication, see Appendix 2.

### Health care resource utilization and sick leave days

Health care resource utilization (HCRU) consisted of both total as well as disease specific visits, and these were tracked separately. For disease specific visits G43* was required as a diagnosis for the visit. All-cause and migraine related values present the minimum and maximum of the HCRU in migraine patients, and most likely the true disease-related HCRU is somewhere in between.

All HCRU data is presented as the absolute value, per patient (cumulative number of events/number of patients), and per patient-year (cumulative number of events/cumulative follow-up). Hence, data in patient-years is adjusted for differences in follow-up, that otherwise would bias those with long follow-up to likely have more HCRU.

### Quartiles of HCRU and sick leaves

To assess the impact of migraine in subjects with high HCRU, migraine patients were divided into quartiles based on either overall visits or sick-leaves in patient-years. For each patient, the total number of visits and sick-leave days were defined and divided by the total patient-wise follow-up time, where after the cohort was divided into quartiles. Patients with less than 6 months of follow-up were excluded from these analyses. Patients in each quartile were characterized as previously, and the corresponding cut-off values for per patient-year estimate for visits and sick leave days were reported.

### Comorbidities

The frequency of comorbidities was assessed at the end of follow-up from ICD-10 codes, in the subgroups and per prophylactic treatment line. Of special interest were depression (F32 single episode, F33 recurrent) and anxiety (F41). The other comorbidities were selected based on highest frequency in the data. A table of the frequency of comorbidities in controls and the primary cohort is presented in Appendix 3.

### Statistical methods

For categorical variables Chi square test was used to test for differences in frequencies (quartiles and comorbidities). For continuous variables (age, HCRU, and follow-up time) Kruskal Wallis test was used to evaluate differences between groups. Otherwise data is descriptive in nature.

## Results

**Fig. 2:**
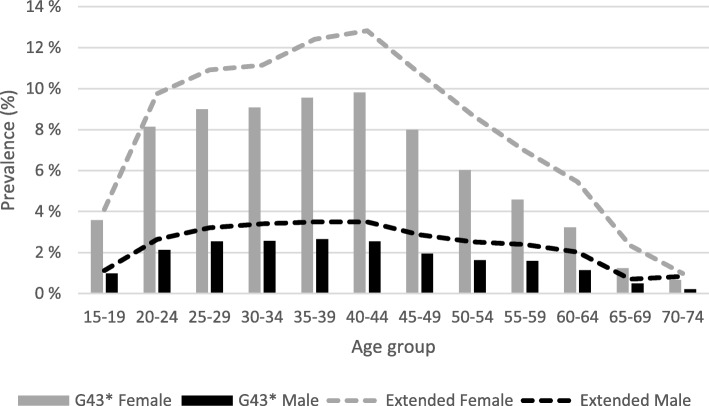
Prevalence of migraine by age group and gender in the occupational health care register. Migraine was defined as a person having G43*at least once (primary cohort, bars), or one of the following: G43*, triptan prescription, any prescription with migraine in the text field (=ext., extended cohort, lines). Female patients (grey), male patients (black)

In the 369,383 individuals with occupational health care data, the overall migraine prevalence was 4.8% in the primary cohort and 6.3% in the extended cohort. Migraine was more frequent in females vs. males (7.4% vs. 2.1%) in the primary cohort, and 9.7% vs. 2.8% correspondingly in the extended cohort. The prevalence of migraine increased between the age of 15 and 20 stabilizing at 8–9% in females and 2.5% in males of the primary cohort and at 10–13% in females and 3.5% in males of the extended cohort in those between 25 and 44 years (Fig. 2). The median age for the first migraine code was 39 years, and 79% of migraineurs were female (Table 1). Migraine with aura was the most common migraine sub-diagnosis code in 2526 persons (14.3%) of the primary cohort.

**Table 1:** Baseline characteristics of consented patients with migraine in the occupational health care register

			Primary cohort subgroups				Failed prophylactic treatments at TT					Botox
		G43, primary cohort	Prophylactic	Acute	No prescription	p	0	1	2	≥3	p	
Follow-up time, median (min-max)		2.84 (0.09–6.00)	3.21 (0.11–6.00)	2.49 (0.09–6.00)	3.05 (0.09–6.00)	< 0.001	2.78 (0.09–6.00)	3.09 (0.11–6.00)	3.79 (0.13–6.00)	4.28 (0.36–6.00)	< 0.001	2.83 (0.09–6.00)
N		17,623	2199	6525	8899	< 0.001	15,424	1767	334	98	< 0.001	53
Female, %		78.9%	81.7%	80.5%	77.0%	< 0.001	78.5%	81.1%	84.4%	83.7%	0.002	83.0%
Age, mean		39 (15–77)	41 (15–70)	38 (15–77)	38 (15–75)	< 0.001	38 (15–77)	41 (15–70)	41 (17–68)	41 (21–61)	< 0.001	39 (20–68)
Prohpylactic prescription, n (%)		2199 (12.5)	2199 (100)	–	–		–	1767 (100)	334 (100)	98 (100)		53 (100)
Acute prescription only, n (%)		6525 (37)	–	6525 (100)	–		6525 (42.3)	–	–	–		–
No prescription, n (%)		8899 (50.5)	–	–	8899 (100)		8899 (57.7)	–	–	–		–
Prophylactic treatment lines	0, n (%)	15,424 (87.5)	–	6525 (100)	8899 (100)		15,424 (100)	–	–	–		–
	1st, n (%)	1767 (10.0)	1767 (80.4)	–	–		–	1767 (100)	–	–		14 (26.4)
	2nd^,^ n (%)	334 (1.9)	334 (15.2)	–	–		–	–	334 (100)	–		18 (34.0)
	3rd, n (%)	98 (0.6)	98 (4.5)	–	–		–	–	–	98 (100)		21 (39.6)
Botulinum toxin A, n (%)		53 (0.3)	53 (0.3)	–	–		–	–	–	–		53 (100)

### Treatments

Only acute migraine drugs were prescribed to 6525 (37%) patients, and at least one prophylactic prescription to 2199 (13%) patients. Moreover, 8899 (51%) individuals with migraine, lacked migraine-related prescriptions in the register (Fig. 1).

Treatment lines were assessed among those with prophylactic prescriptions (Fig. 1). Ninety-eight migraineurs had three or more prophylactic prescriptions, and 53 persons were on botulinum toxin treatment. Patients on prophylaxis were on average 2 years older than those without prophylaxis and the proportion of female gender increased by treatment line (Table 1).

The most common first line prophylactic prescriptions were candesartan (27.4%), followed by amitriptyline (22.1%) and the beta-blockers (cumulative prevalence 27.4%: bisoprolol, propranolol and metoprolol). Second line prophylactic treatments echoed in frequency those of the first line, however topiramate (1st vs. 2nd line: 3.7 vs. 8.1%), nortriptyline (3.6 vs. 6.3%) and nebivolol (1.7 vs. 2.8%) prescriptions were increased (Appendix 1).

### Health care resource utilization and sick leave days

**Fig. 3:**
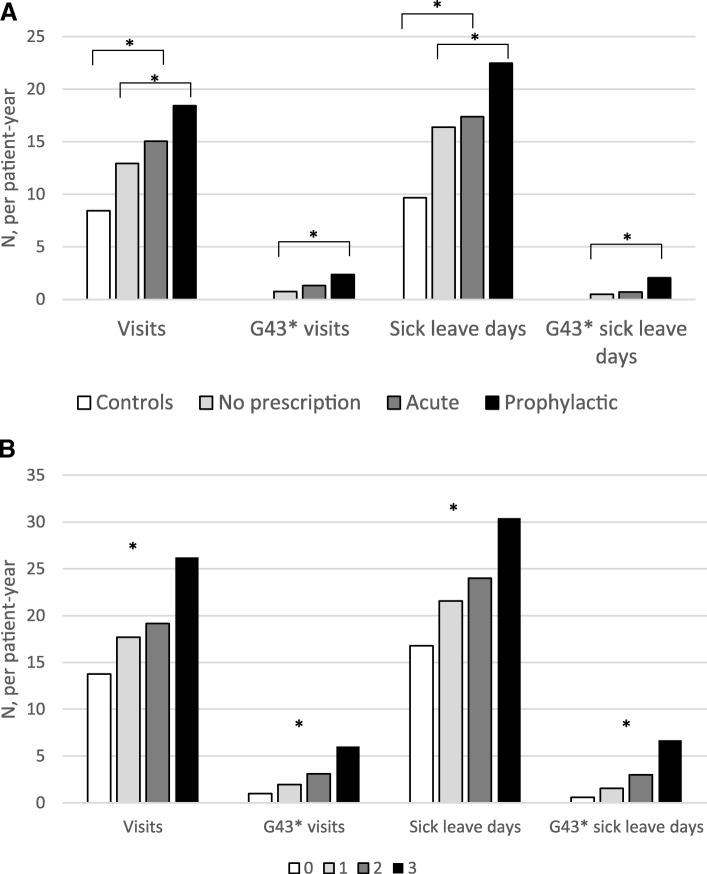
**a** Overall and disease specific (G43*) visits and sick leave days per patient-year in controls (white) and the primary cohort [no prescriptions (grey), acute prescriptions (dark grey), prophylactic prescriptions (black)]. Significance in indicated between controls and migraine patients, as well as between migraine groups. **b** Overall and migraine specific visits and sick leave days per patient-year by prophylactic treatment line. No prophylactic prescriptions (white); 1st prophylactic treatment line (light grey); 2nd prophylactic treatment line (dark grey); 3rd or more treatment line (black). Significance is indicated between four groups. *p < 0.001

Migraine specific and all-cause visits as well as sick leave days were assessed. Patients from the primary cohort with a prophylactic migraine prescription had more visits than those with acute prescriptions or no prescriptions (Table 2, Fig. 3). This was reflected both in total visits (prophylactic vs. acute vs. no prescription: 18.4 vs. 15.0 vs. 12.9 visits per patient-year, *p* < 0.001) and migraine specific visits (2.4 vs. 1.3 vs. 0.8 per patient-year, *p* < 0.001), as well as in total sick leave days (22.5 vs. 17.4 vs. 16.4 per patient-year, *p* < 0.001) and migraine specific sick leave days (2.1 vs. 0.7. vs. 0.5 per patient-year, *p* < 0.001), (Table 2 and Fig. 3). Notably, overall visits in the control population compared to the primary cohort were 8.5 vs. 14.4 (*p* < 0.001) and overall sick leave days 9.7 vs. 17.6 (*p* < 0.001) per patient-year (Table 2).

**Table 2:** Total and disease specific visits and sick-leave days of migraine patients and controls

HCRU and sick leaves	Control	G43*, prim. cohort^1^	G43*, primary cohort								
			Prophylactic	Acute	No prescr.^2^	BT treated	BT untreated^3^	Prophylactic treatment line^4^			
								0	1	2	≥3
N	17,623	17,623	2199	6525	8899	53	17,570	15,424	1767	334	98
Patient-years	76,804	51,397	7033	17,408	26,955	212	51,184	44,363	5435	1209	390
Average follow-up time per patient (years)	4.4	2.8	3.2	2.7	3.0	4.01	2.91	2.9	3.1	3.6	4.0
Visits											
total	649,362	739,803	129,616	261,872	348315*	6480	733323*	610,187	96,241	23,153	10222*
per patient	36.9	42.0*	58.9	40.1	39.1*	122.3	41.7*	39.6	54.5	69.3	104.3*
per patient-year	8.5	14.4*	18.4	15.0	12.9*	30.5	14.3*	13.8	17.7	19.2	26.2*
G43* visits											
total		59,937	16,667	23,013	20257*	1862	58075*	43,270	10,597	3729	2341*
per patient		3.4	7.6	3.5	2.3*	35.1	3.3*	2.8	6	11.2	23.9*
per patient-year		1.2	2.4	1.3	0.8*	8.8	1.1*	1.0	2.0	3.1	6.0*
Sick leave days											
total	742,162	902,454	158,092	302,586	441776*	7845	894609*	744,362	117,207	29,026	11859*
per patient	42.1	51.2*	71.9	46.4	49.6*	148.0	50.9*	48.3	66.3	86.9	121.0*
per patient-year	9.7	17.6*	22.5	17.4	16.4*	37.0	17.5*	16.8	21.6	24.0	30.4*
G43* sick leave days											
total		40,432	14,476	12,384	13572*	2529	37903*	25,956	8265	3613	2598*
per patient		2.3	6.6	1.9	1.5*	47.7	2.2*	1.7	4.7	10.8	26.5*
per patient-year		0.8	2.1	0.7	0.5*	11.9	0.7*	0.6	1.5	3.0	6.7*
Sick leave periods											
total		126,816	19,583	45,800	61433*	907	125909*	107,233	14,576	3551	1456*
per patient		7.2	8.9	7.0	6.9*	17.1	7.2*	7.0	8.3	10.6	14.9*
per patient-year		2.5	2.8	2.6	2.3*	4.3	2.5*	2.4	2.7	2.9	3.7*
G43 sick leave periods											
total		12,362	2776	4754	4832*	260	12102*	9586	1750	626	400*
per patient		0.7	1.5	0.7	0.5*	4.9	0.7*	0.6	1.0	1.9	4.1*
per patient-year		0.20	0.39	0.27	0.18 *	1.22	0.24*	0.22	0.32	0.52	1.03*

*BT* botulinum toxin; Significance indicated between 1: controls and G43* primary cohort; 2: prophylactic, acute and no migraine prescription groups; 3: Botulinum toxin treated and untreated patients; 4: between prophylactic treatment lines

*p < 0.001

Botulinum toxin A treatment was associated with 7.8 times more migraine related visits compared to the rest of the primary cohort (*p* < 0.001, Table 2). Also, a 2.1-fold increase in migraine related sick leave days was found in subject with botulinum toxin prescriptions. Notably, these persons were annually on sick leave 37 days per patient-year and visited the health care provider every twelfth day (31 times per patient-year), indicating an increase in total morbidity associated with chronic migraine treated with botulinum toxin A (Table 2).

An increase in prophylactic treatment lines was associated with an increase in overall visits (no prophylactic prescriptions, vs. ≥3 prophylactic treatments: 13.8 vs. 26.2 visits per patient-year) as well as disease specific visits (no prophylactic prescriptions vs. ≥3 prophylactic treatments: 1.0 vs. 6.0 visits per patient-year), Table 2 and Fig. 3. A comparable increase in sick-leave days was observed: overall sick leave days (no prophylactic prescriptions, vs. ≥3 prophylactic treatments: 16.8 vs. 30.4 sick leave days per patient-year) and disease specific sick leave days (no prophylactic prescriptions, vs. ≥3 prophylactic treatments: 0.6 vs. 6.7 per patient-year). Sick leave periods followed the same pattern (Table 2).

To explore the impact of migraine on overall visits and sick leave days, patients were stratified to quartiles. A shift in prescriptions towards prophylaxis and an increase in prophylactic treatment lines was associated with an increase in overall visits. Notably, of those with > 19.8 visits per patient-year (q4), 20% were on prophylaxis compared to 7.3% of those with less than 7.0 visits per patient-year (q1, Appendix 4). Overall sick-leave days showed a similar increase of prophylactic medication increasing by each quartile of sick-leave days. In those with > 19.9 sick-leave days per patient-year (q4), 16% of patients were on prophylaxis, whereas in patients with less than 1.9 sick-leave days per patient-year (q1) 11% were on prophylaxis (Appendix 5).

### Comorbidities

**Fig. 4:**
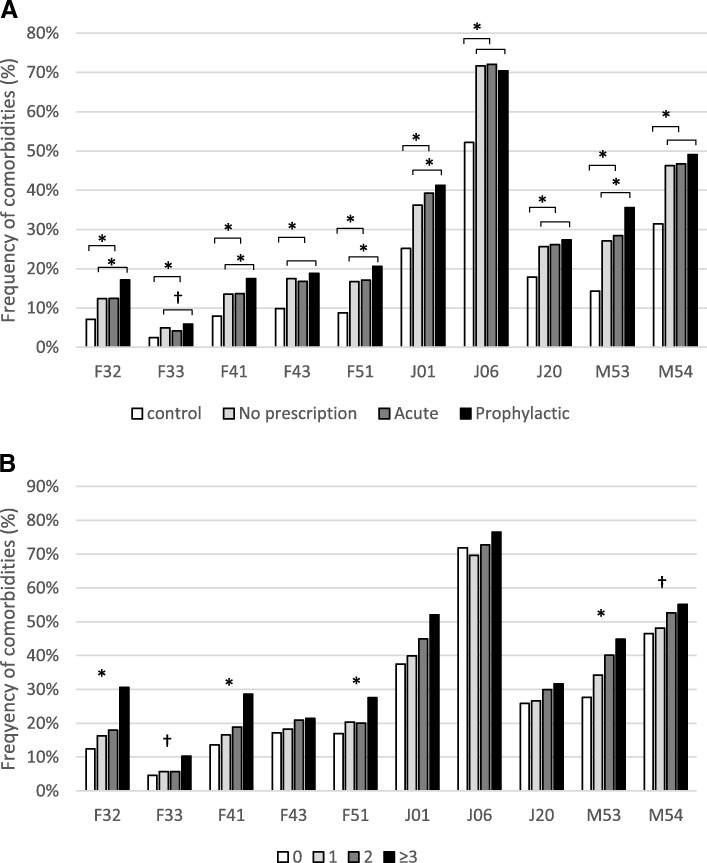
**a** The frequency of comorbidities in controls (white) and patients with migraine (G43*) according to prescriptions of migraine medications [no prescription (light grey), acute (dark grey), prophylactic prescriptions (black)]. Significance is indicated between controls and migraine patients, as well as between migraine groups. **b** Comorbidities by prophylactic treatment line [no prophylactic prescriptions (white); 1st prophylactic treatment line (light grey); 2nd prophylactic treatment line (dark grey); 3rd or more treatment line (black)]. Significance is indicated between four groups. *p < 0.001; † < 0.005. F32 – depression single episode; F33 – depression recurrent; F41 – anxiety; F43- stress reaction; F51 – sleep disorder; J01- sinusitis; J06 – upper respiratory infections; J20 – bronchitis; M53 – dorsopathy; M54 dorsalgia

Those with prophylactic prescriptions had a 1.4-fold increase in the frequency of depression compared to migraineurs with acute prescriptions (F32: 12.5% vs. 17.1%, *p* < 0.001; F33: 4.2% vs. 5.9%, *p* < 0.05). Similarly, 1.3-fold increase of anxiety (F41: 16.8% vs. 18.8%, *p* < 0.001), and 1.2-fold increase in sleep disorders (F51) was observed in patients on prophylaxis (Fig. 4). In the controls these comorbidities were less frequent, 7% had a single episode of depression (F32), 2% recurrent depression (F33), 8% anxiety (F41), and 8.8% sleep disorders (F51) (Fig. 4). Notably, all comorbidities were 1.4–2.0-fold more frequent in migraine patients compared to controls and the difference was more pronounced in those with prophylactic prescriptions (Fig. 4).

There was a positive association between treatment lines and the frequency of comorbidities. Among migraineurs with prophylactic prescriptions, single episode depression (F32) increased in those with no prophylactic prescriptions vs. 3rd prophylactic treatment line from 12.4% to 30.6%; depression recurrent episodes (F33) from 4.6% to 28.6%; and anxiety (F41) from 13.6% to 28.6%, see Fig. 4. A similar trend was also observed for other comorbidities.

## Discussion

In this extensive registry-based retrospective study using EMRs of Finland’s largest private occupational health care provider, 17,623 migraine patients with G43-diagnosis were identified. In this data migraine was associated with substantial morbidity evident as increased comorbidities, as well as increased health care visits and sick leave days when compared to controls. The burden of disease also increased with each additional prophylactic treatment line. The results are aligned with previous studies investigating migraine and indicate that the burden of migraine can be detected in EMRs collected as a part of routine clinical practice.

Treatment unresponsive or refractory migraine, detected as increased numbers of prescribed prophylactic treatments, was associated with up to 3.1-fold increase in all-cause sick leaves and visits compared to controls. Migraineurs on prophylactic treatment were on average on sick leave 22 days annually whereas patients on botulinum toxin A for an average of 37 days annually. The corresponding number of sick-leave days was only ten for the control group, highlighting the increased burden associated with migraine, as lack of migraine was the main difference between these groups. The reason for prophylactic medication switches may in addition to treatment unresponsiveness have been caused by adverse events. The possibility of add-on therapies due to lack of efficacy can neither be excluded.

Directly migraine-related visits and official sick-leave days only present a minor part of true disease burden and are in line with previous findings. In the Eurolight study, the annual per person cost of migraine-related absenteeism was 371€ [26], which would correspond to approximately 2 days of sick leave in Finland. Similar annual migraine related absenteeism days were found in a longitudinal survey in the US [27]. In our study, botulinum toxin treatment was associated with 11.9 migraine related sick-leave days per patient-year, also in line with approximately 10 days previously reported [27]. However, the effect of prophylactic treatment lines on disease burden has been poorly investigated and the incremental increase of all-cause sick-leaves and visits per additional prophylactic treatment line, underlines that migraine related disease burden cannot solely be assessed by migraine related visits or sick-leaves, but should be considered in a larger perspective. Moreover, this study also investigated the migraine burden per se compared to age and gender matched controls, where all-cause sick-leaves were 1.8 and visits 1.7 more frequent in the migraine population than in the controls per patient-year.

The increase in comorbidities for migraine patients when compared to controls highlights an increased morbidity in this group. This was further supported by the positive correlation of treatments lines and frequency of comorbidities. In patients with 3 or more prophylactic treatments, a mental disorder such as major depressive disorder or anxiety, was diagnosed in every third person. This contrasts with controls where depression was diagnosed in every 14th (7%) person and anxiety in every 13th (8%) person.

The previously reported frequency of comorbidities varies partly because of methodological aspects. In chronic migraine up to 57% of patients were detected to have major depression and 8% anxiety when screened in a clinic-based study [28]. Also, an increase of prophylactic medication in subjects with migraine and depression, compared to migraine alone, has been described even though prophylactic treatment lines were not assessed [29]. Depression and migraine are bidirectionally associated, with both illnesses adding to the risk of developing the other [30]. Common neurological etiology is also shared between migraine, depression and sleep disorders [31], which in addition to reaction to severe stress and dorsopathies, were more frequent in patients with migraine compared to controls.

Migraine has been one of the top five leading causes for years lived with disability (YLD) globally since 1990, highlighting the unmet need of more effective migraine management [2]. This was also seen in our study in the concomitant increase between prophylactic treatment lines and absenteeism, visits as well as comorbidity. Multimorbidity is known to influence the overall disease burden due to polypharmacy, reduced quality of life, increased disability and mortality as well as increased HCRU. Multimorbidity is thought to include 2–3 or more simultaneous conditions and has been described in chronic migraine [32]. The higher frequency of depression and anxiety, accompanied by the seven most common comorbidities in migraine patients in the study, points at the possibility of notable existence of multimorbidity also in less severe migraine patients. Moreover, efficient treatment of migraine including psychiatric comorbidities such as depression is considered essential in avoiding migraine chronification [14].

Diagnosing people with migraine is important in the light of making optimal treatment decisions and to positively influence the disease burden. According to previous mostly survey- based prevalence studies, about 9.6–15% among the general population suffer from migraine [25, 33–35]. Therefore, the total migraine prevalence of 4.8–6.3% among people in occupational health care reported here would seem very conservative, especially when considering the well-known age-dependence in migraine prevalence [36]. However, it must be noted that the prevalence has been extracted from EMRs, collected as a part of routine clinical care plausibly explaining the lower numbers [37]. The prevalence for female were 7.4–9.7% and for male 2.1–2.8% with a gender ratio of 3.5. These estimates are similar to the ones previously found [24, 36]. Also, some selection bias may be seen in the prevalence figures due to the restriction of analyses to those with occupational health care, leaving unemployed, students and those incapacitated to work outside of the study. In addition, some patients with milder forms of migraine may have been excluded due to lack of diagnosis or need for intensified medical management.

Survey-based studies further show that up to 44% of migraine patients never receive a medical diagnosis [34, 38], and thus approximately half of the patients could be expected to have migraine diagnosis recorded in EMR. Moreover, of patients with a clinical diagnosis of primary non-migraine headache, 43% were reassessed in a study to have migraine based on longitudinal follow-up data [39]. We also found that approximately 5700 or 24% of patients were likely misdiagnosed lacking a migraine ICD-10 code even if they were prescribed triptans or other migraine-relevant medications in combination with migraine written in the free text field of the prescription.

This study included some limitations. Patients were divided into subgroups and treatment lines based on prescribed medication at the health care provider. It cannot be excluded that the subjects may have visited other health care instances for migraine care i.e. public hospitals for ER visits, and migraineurs may have additionally utilized over the counter drugs that are not included in any register. Yet, the restriction of the study cohort to those with occupational health care was done to minimize this bias, and to be able to assess the disease burden affecting working population in Finland. The prophylactic cohort was extracted based on strict search criteria, and it is likely that some patients with G43 diagnosis and prescriptions with the chosen prophylactic treatment ATC-codes without migraine in the free text field may have been on migraine prevention. Based on this, the true prophylaxis prevalence is likely more than the detected 13% in our study. On the other hand, the proportions of prescriptions for migraine detected here do not appear low when compared to the recent estimates from the Eurolight study [40]. Moreover, monthly migraine days are not routinely recorded in the EMR or included in ICD-10, and thus the separation of migraine into EM and CM was not possible in this study. Therefore, patients on botulinum toxin A were the only confirmed CM patients.

It is also noteworthy that migraine and all-cause related absenteeism is underestimated in this study. First, short 1–3-day absences can be reported with self-notice and do not end-up in the occupational health care records that were examined in this study. Secondly, sick-leaves are linked to diagnosis in the EMR only when a patient sees a health care professional at the provider. It has been estimated that absenteeism is only responsible for one third of migraine-related indirect costs and presenteeism is thought to account for the rest two thirds [26]. Estimations of presenteeism were not included in this study. Further studies are warranted to elucidate whether self-notice absenteeism and presenteeism correlate with the increased disease burden detected here.

Strengths of the study were that data was gathered in a real-world setting without strict inclusion/exclusion criteria, and the ability to put the disease burden into perspective when compared to those not suffering from migraine. The study included EMRs of the Finland’s largest private occupational health care provider having more than 150 clinics across the country. As occupational health care must be provided by the employer according to the Finnish law, there is also no selection of members due to societal status or position from the health care provider. Further, the study examined true clinical data and is not claims based. As headache diaries are not included in EMRs, the lack of successful migraine management and more severe disease was estimated through history of one or more prophylactic treatment failures for migraine prevention.

The study uniquely combines prescriptions, comorbidities and absenteeism in patients able to work and controls to assess the impact of failed treatments on increasing disease burden. It is possible that failed treatments leading to increased disease burden may reflect chronification of migraine. Another interesting aspect in the future would be to evaluate the impact of successful migraine prevention on disease burden. There has been at least one study showing a potential concomitant positive influence on depression and anxiety when chronic migraine was treated with botulinum toxin A [17, 41].

## Conclusions

This study provided new evidence on the disease burden of migraine from EMRs collected as a part of routine clinical care in Finland and complements the extensive evidence mostly from survey-based studies. In this study, migraine patients show a substantially higher extent of comorbidities, sick-leave days and health care visits compared to their age- and gender-matched counterparts. Moreover, the increased disease burden was not directly linked to G43-diagnosis but was rather detected via other diagnostic codes potentially reflecting other migraine-related comorbidity. This study highlights the need to more effectively manage migraine to avoid detrimental effects on health and quality of life that are associated with treatment unresponsive or undertreated migraine. Unresponsive or undertreated migraine, evidenced by an increase in failed treatment lines, increases morbidity and inevitably leads to productivity losses that pose an increased burden on the health care system and society.

## Appendix 1

**Table 3:** Prophylactic treatment lines of patients with G43*diagnosis and the word migraine in the free text of a prophylactic migraine prescription. A new treatment line was defined as a new drug prescription

		Prophylactic treatment lines							Prescr, by neurologist (%)
		1 (%)	2 (%)	3 (%)	4 (%)	5 (%)	6 (%)	7 (%)	
ATC	Drug name	*N* = 2199	432	98	28	10	2	1	
C09CA06	Candesartan	27.38	23.61	21.43	7.14	20.00			22
N06AA09	Amitriptyline	22.06	16.90	14.29	3.57	10.00			17
C07AB07	Bisoprolol	15.73	10.19	10.20	3.57				15
C07AA05	Propranolol	6.82	4.17	4.08	14.29	30.00			17
C07AB02	Metoprolol	4.91	4.86	5.10	7.14				6
N06CA01	Amitriptyline and psycholeptics	4.68	3.47	4.08	7.14		50.00		47
N03AX11	Topiramate	3.73	8.10	7.14	7.14	20.00		100	41
N06AA10	Nortriptyline	3.55	6.25	4.08					42
C07AB12	Nebivolol	1.68	2.78	1.02	3.57				22
M03AX01	Botulinum toxin	1.05	3.70	11.22	7.14	10.00			64
C08DA01	Verapamil	1.05	1.62		3.57				23
N03AX09	Lamotrigine	0.86	0.93	3.06					50
N03AG01	Valproic acid	0.82	3.70	1.02	7.14		50.00		58
C09CA01	Losartan	0.82	1.62	1.02					4
C09CA08	Olmesartan medoxomil	0.64	1.16	1.02					80
N03AX12	Gabapentine	0.50	1.16	2.04					56
C09CA07	Telmisartan	0.45	0.23						9
G03CA03	Estradiol	0.36	0.23						0
N06AA12	Doxepin	0.32	0.69		3.57				64
N03AX16	Pregabaline	0.27	0.69						11
C07AB03	Atenolol	0.23							40
N06AA06	Trimipramine	0.18	0.23	1.02					67
G03 AC09	Desogestrel	0.18	0.23		3.57				0
N06AX21	Duloxetine	0.14	0.69	1.02					29
N06AX16	Venlafaxine	0.14	0.23		3.57				100
G02BA03	Plastic IUD with progestogen	0.14		1.02		10.00			0
C09DA06	Candesartan and diuretic	0.09	0.23		3.57				0
C08CA06	Nimodipine	0.09	0.23						100
C08CA05	Nifedipine	0.09		1.02	3.57				0
C08CA01	Amlodipine	0.09							0
C07BB07	Bisoprolol and thiazides	0.09							0
N06AB10	Escitalopram	0.09							50
G03AA12	Drospirenone ja ethinyliestradiol	0.05	0.69						0
C08DB01	Diltiazem	0.05	0.23						0
G03HB01	Cyproterone and estrogen	0.05	0.23						0
N03AE01	Clonazepam	0.05		1.02					50
C07AB05	Betaxolol	0.05							0
C07AA03	Pindolol	0.05							0
N06AX11	Mirtazapine	0.05							0
N06AX22	Agomelatine	0.05							0
G03AA14	Nomegestrol and estradiol	0.05							0
N06AB03	Fluoxetine	0.05							0
C09AA05	Ramipril	0.05							100
C09BX01	Perindopril, amlodipine and indapamide	0.05							0
C07BB02	Metoprolol and thiazides	0.05							0
N03AF02	Oxcarbazepine	0.05							0
G03BA03	Testosterone	0.05							0
C07AA07	Sotalol	0.05							100
G02BB01	Vaginal ring with progestogen and estrogen	0.05							0

## Appendix 2

**Table 4:** Acute migraine prescription frequency on the G43* population, the list includes acute medication from both acute and prophylactic groups (*N* = 8244)

ATC	Medication	Group	N	Proportion of patients (%)
N02CC01	Sumatriptan	Selective serotonin (5HT1) agonists	3962	48.06%
M01AE02	Naproxen	Antiinflammatory and antirheumatic products	1698	20.60%
N02CC07	Frovatriptan	Selective serotonin (5HT1) agonists	1227	14.88%
N02CC04	Ritsatriptan	Selective serotonin (5HT1) agonists	989	12.00%
M01AE01	Ibuprofen	Antiinflammatory and antirheumatic products	889	10.78%
N02CC03	Zolmitriptan	Selective serotonin (5HT1) agonists	763	9.26%
N02CC06	Eletriptan	Selective serotonin (5HT1) agonists	673	8.16%
A03FA01	Metoclopramide	Propulsives	664	8.05%
N02CC05	Almotriptan	Selective serotonin (5HT1) agonists	566	6.87%
M01AG02	Tolfenamic acid	Antiinflammatory and antirheumatic products	530	6.43%
N02BE01	Paracetamol	Antiinflammatory and antirheumatic products	391	4.74%
M01AE03	Ketoprofen	Antiinflammatory and antirheumatic products	192	2.33%
N02AA59	Codein combnations	Opioids	178	2.16%
M01AB05	Diclofenac	Antiinflammatory and antirheumatic products	165	2.00%
N02CC02	Naratriptan	Antiinflammatory and antirheumatic products	107	1.30%
H02AB06	Prednisolone	Corticosteroids for systemic use, plain	95	1.15%
M01AE17	Dexketoprofen	Antiinflammatory and antirheumatic products	70	0.85%
N02AJ06	Codeine and paracetamol	Opioids	61	0.74%
H02AB04	Methylprednisolone	Corticosteroids for systemic use, plain	48	0.58%
N02BA01	Acetylsalicylic acid	Other analgesics and antipyretics	43	0.52%
M01AB01	Indometacin	Antiinflammatory and antirheumatic products	38	0.46%
M03 BC51	Orfenadrine combinations	Muscle relaxants, centrally acting agents	38	0.46%
M01AH05	Etoricoxib	Antiinflammatory and antirheumatic products	26	0.32%
M03BX02	Tizanidine	Muscle relaxants, centrally acting agents	24	0.29%
M01AG01	Mefenamic acid	Antiinflammatory and antirheumatic products	21	0.25%
A04AA01	Ondansetron	Serotonin (5HT3) antagonists	16	0.19%
H02AB07	Prednisone	Corticosteroids for systemic use, plain	14	0.17%
N02AX02	Tramadol	Opioids	14	0.17%
H02AB02	Dexametasone	Corticosteroids for systemic use, plain	13	0.16%
M01AE52	Naproxen and Esomeprazole	Antiinflammatory and antirheumatic products	12	0.15%
A03DA02	Pitofenone and Analgesics	Synthetic anticholinergic agents in combination with analgesics	8	0.10%
M01 AC06	Meloksicam	Antiinflammatory and antirheumatic products	5	0.06%
M03 BC01	Orfenadrine	Muscle relaxants, centrally acting agents	5	0.06%
N02AJ08	Codein and ibuprofen	Opioids in combination with non-opioid analgesics	4	0.05%
M01AH01	Celecoxib	Antiinflammatory and antirheumatic products	3	0.04%
N02BE51	Paracetamol combinations	Antiinflammatory and antirheumatic products	3	0.04%
M01AB08	Etodolac	Antiinflammatory and antirheumatic products	1	0.01%
M01AX01	Nabumetone	Antiinflammatory and antirheumatic products	1	0.01%
M03BX01	Baclofen	Muscle relaxants, centrally acting agents	1	0.01%
N02AX52	Tramadol combinations	Opioids in combination	1	0.01%
N02BA51	Acetylsalicylic acid, combinations	Other analgesics and antipyretics	1	0.01%

## Appendix 3

**Table 5:** The frequency (%) of comorbidities in controls and patients of the primary cohort

		Major depressive disorder, single episode	Major depressive disorder, recurrent	Other anxiety disorders	Reaction to severe stress, and adjustment disorders	Sleep disorders not due to a substance or known physiological condition	Acute sinusitis	Acute upper respiratory infections of multiple and unspecified sites	Acute bronchitis	Other and unspecified dorsopathies, not elsewhere classified	Dorsalgia
		F32	F33	F41	F43	F51	J01	J06	J20	M53	M54
Cohort	Control	7.1	2.5	7.9	9.9	8.8	25.2	52.2	17.9	14.3	31.4
	G43* primary cohort	13.0	4.8	14.1	17.4	17.4	38.0	71.7	26.0	28.6	46.8
	*p*-value	< 0.001	< 0.001	< 0.001	< 0.001	< 0.001	< 0.001	< 0.001	< 0.001	< 0.001	< 0.001
Primary cohort subgroups	No prescription	12.4	5.0	13.5	17.5	16.7	36.2	71.7	25.6	27.1	46.3
	Acute prescriptions	12.5	4.2	13.7	16.8	17.1	39.2	72.1	26.1	28.4	46.7
	Prophylactic prescription	17.1	5.9	17.5	18.8	20.6	41.2	70.4	27.3	35.6	49.1
	*p*-value	< 0.001	0.002	< 0.001	0.091	< 0.001	< 0.001	0.319	0.242	< 0.001	0.058
Prophylactic treatment line	0	12.4	4.6	13.6	17.2	16.9	37.5	71.8	25.8	27.6	46.5
	1	16.2	5.7	16.6	18.3	20.3	39.9	69.6	26.6	34.2	48.1
	2	18.0	5.7	18.9	21.0	20.1	44.9	72.8	29.9	40.1	52.7
	≥3	30.6	10.2	28.6	21.4	27.6	52.0	76.5	31.6	44.9	55.1
	*p*-value	< 0.001	0.011	< 0.001	0.138	< 0.001	< 0.001	0.155	0.182	< 0.001	0.027
Botulinum toxin prescription	no	12.9	4.8	14.0	17.4	17.4	37.9	71.7	26.0	28.6	46.8
	yes	32.1	9.4	28.3	22.6	15.1	54.7	73.6	43.4	52.8	56.6
	*p*-value	< 0.001	0.112	0.003	0.312	0.662	0.012	0.755	0.004	< 0.001	0.152

## Appendix 4

**Table 6:** Overall visits per patient-year of the primary cohort stratified by quartiles

Visits (overall)	q1	q2	q3	q4	*p*-value*
Cut-off (visits per patient-year)	< 7.03	7.03–12.22	12.23–19.79	> 19.79	–
Follow-up time, years (median)	3.25	3.35	3.18	2.64	< 0.001
N	3990	3992	3991	3991	–
Female (%)	73.7%	77.1%	81.5%	83.9%	< 0.001
Age, years (mean)	36.06	39.07	40.14	40.75	< 0.001
Acute prescription only, %	34.1%	34.5%	38.2%	38.8%	< 0.001
No prescription, %	58.6%	54.0%	48.6%	41.9%	
Prophylactic prescription, %	7.3%	11.5%	13.2%	19.3%	
Botulinum toxin A, %	0.1%	0.1%	0.3%	0.9%	< 0.001
Prophylactic treatment line 0	92.68%	88.53%	86.82%	80.68%	< 0.001
1	6.64%	9.17%	10.52%	14.48%	
2	0.65%	2.00%	2.08%	3.36%	
≥3	0.03%	0.30%	0.58%	1.48%	

**P*-value for test for all quartiles equal

## Appendix 5

**Table 7:** Overall sick leave days per patient-year of the primary cohort stratified by quartiles

Sick leave days (overall)	q1	q2	q3	q4	*p*-value*
Cut-off (sick leave days per patient-year)	< 1.92	1.93–7.09	7.10–19.85	> 19.85	–
Follow-up time, years (median)	2.84	3.28	3.30	3.06	< 0.001
N	3990	3992	3991	3991	–
Female, (%)	73.0%	79.3%	81.5%	82.5%	< 0.001
Age, years (mean)	39.9	38.4	38.2	39.5	< 0.001
Acute prescription only, %	36.3%	36.5%	36.1%	36.6%	< 0.001
No prescription, %	53.1%	51.6%	50.8%	47.7%	
Prophylactic prescription, %	10.7%	11.9%	13.1%	15.7%	
Botulinum toxin A, %	0.2%	0.1%	0.3%	0.7%	< 0.001
Prophylactic treatment line 0	89.3%	88.1%	86.9%	84.3%	< 0.001
1	9.1%	9.6%	10.2%	11.9%	
2	1.3%	2.0%	2.0%	2.8%	
≥3	0.3%	0.3%	0.8%	1.0%	

**P*-value for test for all quartiles equal

## Abbreviations

CM: Chronic migraine
EM: Episodic migraine
EMR: Electronic medical records
HCRU: Health care resource utilization
